# Service evaluation and research project: pros and cons of centralisation of ECT services in Pennine Care NHS Foundation Trust

**DOI:** 10.1192/bjo.2021.880

**Published:** 2021-06-18

**Authors:** Madhumanti Mitra, Katherine Hayden

**Affiliations:** Pennine Care NHS Foundation Trust

## Abstract

**Aims:**

The aim of this project would be to understand about the pros and cons/ benefits and risks of using a centralised model of ECT (currently being followed due to COVID restrictions) rather than a decentralised model of ECT (which has been the norm for a long time). A comparative account of both types of systems would help identify whether it could be implemented in Pennine Care and the results could be potentially transferable to other similar settings.

**Method:**

An online survey was undertaken from staff members working in Pennine Care who have been involved in ECT delivery and management. The survey was undertaken between 07/12/2020 and 11/01/2021 and included questions relating to pre-COVID ECT (June 2019 to February 2020) and during-COVID ECT (March 2020 to November 2020). Questions were around travel of staff/patients for ECT, whether they had a preference for a model and their reasons around it. Data were summarised in MS Excel and free text comments analysed to gain an understanding of staff's preferences and reasoning behind their choices.

**Result:**

Although some boroughs had patients attending from other boroughs in pre-COVID times, considerably more staff (53.85%) and patients (61.54%) had to travel for ECT during COVID times. Around 50% staff expressed a clear view for decentralised services; the common reasons being safer for patients, better continuity of care, less travelling issues, patients more likely to consent, easier to manage correct paperwork, easier to send staff who knows patients well, less driving for staff and likely less cancellations. Around 40% staff expressed a clear view for centralised services; the common reasons being less staff needed, better infection control, easier to maintain staff skills, efficiency, developing clinical expertise with larger number of cases, education opportunities, better set-up of clinics, transportation and accessibility. Some concerns raised for the centralised model were managing patients with complex anaesthesia, travelling for unwell/ disturbed patients, too high patient numbers, poor communication and impact on training.

**Conclusion:**

In summary, there was a mixed view of which services are preferable. Further discussion in trust wide ECT forum will be helpful to move things forward. Although it is likely that services may shift from a decentralised to a centralised system, we need to ensure this is done safely and in particular, address the main concerns around centralisation.

